# Similar cellular migration patterns from niches in intervertebral disc and in knee-joint regions detected by *in situ* labeling: an experimental study in the New Zealand white rabbit

**DOI:** 10.1186/scrt315

**Published:** 2013-09-02

**Authors:** Helena Barreto Henriksson, Anders Lindahl, Eva Skioldebrand, Katarina Junevik, Carolina Tängemo, Johan Mattsson, Helena Brisby

**Affiliations:** 1 Department of Orthopaedics, Institute of Clinical Sciences, Sahlgrenska University Hospital, Gothenburg University, 413 45 Gothenburg, Sweden; 2 Department of Clinical Chemistry and Transfusion Medicine, Institute of Biomedicine, Sahlgrenska University Hospital, Gothenburg, Sweden; 3 Centre for Cellular Imaging, the Sahlgrenska Academy, University of Gothenburg, Gothenburg, Sweden; 4 Department of Microbiology and Immunology, Institute of Biomedicine, Sahlgrenska University Hospital, Gothenburg, Sweden

## Abstract

**Introduction:**

Potential stem cell niches (SNs) were recently reported in intervertebral discs (IVDs) and knee joints (KJs) in different mammals (located adjacent to the epiphyseal plate; EP). The aim here was to examine further possible cellular migration and migration directions of cells originating from niches possibly involved in regeneration of cartilaginous tissues in the IVD and in the KJ regions in adult mammals.

**Methods:**

In total, 33 rabbits were used in studies A through C.

A. IVD cells were sorted; fluorescence-activated cell sorting (FACS) by size (forward scatter; ≤10 μm or >10 μm or GDF5^+^ cells (anti-GDF5 antibody). Sorted cells, labeled with cell tracer (carboxyfluorescein-diacetate-succinimidyl ester; CDFA-SE) were applied on IVD explants *in vitro*. Migrating cells/distance was evaluated by fluorescence- and confocal-microscopy (FC).

B. DNA labeling was performed with BrdU (oral administration). Animals were killed (14 to 56 days), KJs collected, and BrdU^+^ cells visualized with immunohistochemistry (IHC)/anti-BrdU antibody in SN and articular cartilage (AC).

C. Cell tracer: (Fe-nanoparticles: Endorem) were injected into SNs of IVDs (LI-LV) and KJs (tibia). Animals were killed after 2 to 6 weeks. Fe-labeled cells were traced by ferric-iron staining (Prussian blue reaction; Mallory method).

**Results:**

A. GDF5^+^ cells and ≤10-μm cells displayed the best migration capability in IVD explants. GDF5^+^ cells were detected at a tissue depth of 1,300 μm (16 days). B. BrdU^+^ cells were observed in early time points in niches of KJs, and at later time points in AC, indicating a gradual migration of cells. C. Fe^+^ cells were detected in IVDs; in annulus fibrosus (AF) in 11 of 12 animals and in nucleus pulposus (NP) in two of 12 animals. In AC (tibia), Fe^+^ cells were detected in six of 12 animals. In the potential migration route (PMR), from niches toward the IVD, Fe^+^ cells (three of 12 animals) and in PMR toward AC (KJs) (six of 12 animals) were detected.

**Conclusions:**

Results indicate similar cellular migration patterns in cartilage regions (IVD and KJs) with migration from stem cell niche areas into the mature cartilaginous tissues of both the KJs and the IVD. These findings of a cellular migration pattern in mature cartilage are of interest from tissue-repair and engineering perspectives.

## Introduction

Musculoskeletal diseases represent a major part of medical costs in the Western society. Cartilage defects in the articular cartilage (AC) of the knee joint (KJ) leads to localized osteoarthritis and degeneration of the intervertebral disc (IVD) [1,2] and are difficult to treat clinically [3-6]. Cartilage in general is considered to lack or to have poor repair capacity because of the avascular tissue structure and poor nutrition of cartilaginous cells provided by diffusion mechanisms [7-9]. Furthermore, the chondrocytes or chondrocyte-like cells are entrapped in the extracellular matrix and have a slow cellular turnover, which contributes to less repopulation of the defect and subsequent regeneration and repair of an injury. In general, articular cartilage has been considered to be a tissue containing one cell type, terminally differentiated chondrocytes. The center part, nucleus pulposus (NP) of a human adult intervertebral disc, contains mainly chondrocyte-like cells surrounded by extracellular matrix, where the most common collagen type is collagen type II and minor fractions of collagen types VI and XI [8,10]. The NP is enclosed by the annulus fibrous (AF), which consists of cells, microfibrillae, and ring-like structures; the lamellae (collagen type I-rich fibers) [11,12]. In addition, previous studies detected the presence of cells expressing progenitor/stem cell markers in adult cartilage [13-16], and cells isolated from articular cartilage of KJs have been demonstrated to possess clone-formation capacity *in vitro*[17,18].

Limited knowledge exists about the normal regeneration processes in cartilaginous tissues, and increased knowledge in this area is of great importance from clinical and tissue-engineering perspectives. The homeostasis of many tissues is regulated by a balance of proliferation and differentiation of adult stem cells [19-21]. In different tissues, stem cells are located in special anatomic structured microenvironments termed niches. The location and nature of these stem cell niches (SNs) can vary, depending on the tissue type [20,22]. SNs have been identified in, for example, skin, bone, intestine, and brain (hippocampus) [23-26]. In the niche, stem cells undergo mitosis in a hypothetical asymmetric cell-division pattern, in which one daughter cell gives rise to a more-differentiated progeny, whereas the other cell maintains the original stem cell stage [19,27,28].

SNs have been proposed to exist in the intervertebral disc region, located adjacent to the epiphyseal plate (EP) and in the outer zone of the AF [29]. In the knee joint, progenitor cells have been detected in the superficial zone of articular cartilage [18], and potential stem cell niches (SNs) have been demonstrated in the synovial membrane [30,31] and in the zone of the Ranvier groove, (adjacent to the EP of the knee joint), which anatomically corresponds to the identified area in the IVD [14,32,33].

Currently, limited knowledge exists about the *in vivo* potential cellular activities, chemoattractants for stem cell recruitment, and migration patterns of progenitor cells originating from these niches (SNs). However, recently in a lapine model, cells that expressed the prechondrocytic markers growth- and differentiation factor-5; GDF5 (GDF5 also termed cartilage-derived morphogenic protein-1); CDMP1 and bone-morphogenic-protein-14; BMP14), SOX9 (sex-determining region Y-box) and, for example, Snail homolog 1, a cytosol protein that is involved in cellular migration, were reported around these niches (SNs) in the IVD region [29]. The chondrogenic lineage marker GDF5 was chosen for the explant studies in the present study because of its role in joint-development and cartilage-regeneration mechanisms [34-36]. In previous studies, it was demonstrated that injection of GDF5 into injured/degenerated IVDs resulted in some positive effects on disc height, improvement in magnetic resonance imaging scores [37] (bovine and lapine models), and extracellular matrix accumulation [38] (*in vitro* study, human cells), as well as in *in vivo* studies (murine model) [39].

Detection of cell-proliferation rates as well as slow-cycling cells (for example, stem cells) within different tissues can be performed with a commonly used *in vivo* labeling technique by using 5-bromo-2-deoxyuridine (BrdU) [22,40]. BrdU is a thymidine analogue, incorporated into proliferating cells during mitosis [22,27,40,41] when administered, and can thereafter be detected by using antibodies directed toward the BrdU molecule. Cells that do not undergo mitosis during the BrdU exposure period incorporate no BrdU into their DNA. Hence, after the exposure period, the amount of DNA-incorporated BrdU decreases in the labeled cells with each mitotic event, until it decreases below detection level. Thus, in tissues that have rapid cell turnover, BrdU will be detected solely at early time points after labeling. Label-retaining (slow-cycling) cells retain BrdU for a longer period in, for example, the stem cell niches [14,22,42], as well as during migration from these niches. These cells can be traced with BrdU-labeling methods [26,29,43]. Previously, such migration was reported (lapine model) in the IVD region by using BrdU method, in which a shift of the BrdU^+^ labeled fraction of cells was observed in different locations, indicating a gradual cellular migration toward the IVD. Sparse BrdU was observed within the AF and NP [29,42].

Epithelial-mesenchymal transition (EMT) is an evolutionarily well-conserved process, present in many types of organisms, and means that cells dissolve from a certain tissue region and migrate to different locations [44,45]. EMT is common in many processes, such as activation of immunoreactive cells (for example, in macrophages and in tumor cells that are migrating and developing tumor metastases [46,47]). During the EMT process, the cytoskeleton of the cells is rearranged to a adapt a more flattened migratory phenotype [48,49], and Snail homolog 1, a member of the Snail superfamily proteins [50,51], is one of the key intermediators involved in rearranging events in the cytoskeleton. Different types of fluorochrome compounds can further be used as cell tracers in studies of cellular migration (for example, carboxyfluorescein diacetate or succinimidyl ester (CDFA-SE)). CDFA-SE passively diffuses over the cell membrane and is nonfluorescent until acetate groups of CDFA-SE are cleaved by intracellular esterases to form fluorescent, amine-reactive carboxyfluorescein succinimidyl esters. The succinimidyl ester group reacts with intracellular amines, and fluorescent conjugates are created and well retained within the cytosol.

For tracing of migrating cells, nontoxic iron particles (for example, super-paramagnetic nanoparticles (SPIOs, Endorem (Guerbet, Villepinte, France))) can be used for the detection of tumor metastases in the lymphatic system and for labeling of cells in *in vitro* and *in vivo* experiments [52-54].

The aim of the present study was to address the hypothesis of possible cellular migration and migration directions of cells originating from niches potentially involved in tissue maintenance and regeneration of cartilaginous tissues in the intervertebral disc and in the knee-joint regions in adult mammals.

## Materials and methods

### Animals

In total, 33 female New Zealand white rabbits were used in the study. For *in situ* labeling with cell tracers (*n* = 12) and *in vivo* BrdU labeling (*n* = 11), the animals were 3 months old, and for the IVD explant study (*n* = 10), the animals were 11 months old. The animals demonstrated good health status and weight gain during the entire experiment time. All animal experiments were approved by the regional animal ethics committee, Vastra Gotaland region, Sweden (ethical permissions numbers 262–2010 and 338–2007).

### Experimental design

**Figure 1:**
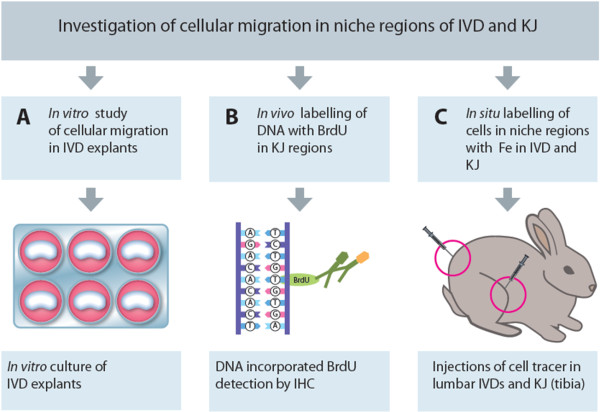
**A schematic overview of the experimental design. (A)** A novel experimental assay with fluorochrome-labeled cells to study cellular migration through IVD explants *in vitro* was developed. **(B)** A study with *in vivo* labeling of DNA with BrdU to examine cell tracing and cell proliferation in different regions of KJs. In addition, immunostaining for SNAI1 was performed. **(C)***In situ* labeling of cells with fluorochrome and iron nanoparticles for tracing of migrating cells from niches in the IVD and KJ regions.

Three main experiments, A through C (Figure 1) were performed to investigate cellular migration in niche areas (SNs) of the IVD and KJ (the zone of Ranvier groove).

### Preparation of tissues for histology analyses

The tissues were placed in 4% formaldehyde (Histofix, Gothenburg, Sweden), decalcified by using a 12.5% EDTA solution, and imbedded in paraffin for sectioning for microscopy analyses. Paraffin sections (5 to 7 μm) were deparaffinized with xylene, 2 × 10 minutes, and rehydrated in 99%, 95%, and 70% ethanol for 5 minutes in each solution before IHC analyses.

#### ***Experiment A***

##### 

**Figure 2:**
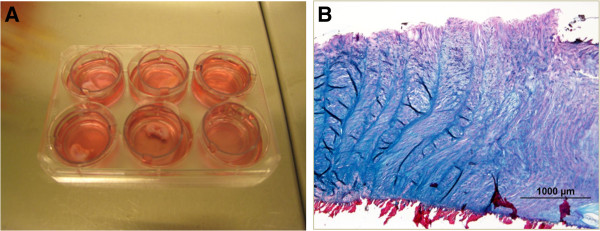
**Cell cultures and morphology of IVD explants. (A)** Image of lapine IVD explants; whole IVDs were used and cultured in cell-culture inserts in six-well plates. **(B)** Histology image of a lapine IVD explant. Staining: Alcian Blue Van Gieson (proteoglycans, blue;collagens, red).

**IVD explant cultures and isolation of cells from IVDs for explant assays with flow cytometry** A new *in vitro* system was designed for the study of the migration capability of cells in fibrocartilage and IVD explants. IVD explants were collected from adult rabbits (age 11 to 13 months). A circular mechanical saw was used for collecting the IVDs from the dead animals. At one side of the IVD, the bone was kept intact for stability reasons, and, on the other side, the outer layers of the IVD tissue were exposed and cultured as explants in cell-culture inserts (Becton Dickinson, Franklin Lakes, NY, USA) in six-well plates (Becton Dickinson) (Figure 2A, B).

Two sets of explant studies were performed on IVDs dissected from rabbits (*n* = 10). In the first one, the migration capability of small (<10-μm cell diameter) versus large cells (>10-μm cell diameter) was studied, and in the second series, the migration capability of GDF5-positive cells was examined.

The cells were sorted from the total cell population into two cell populations by size by using the forward-scatter function (FSC) or by an antibody directed toward GDF5 of the IVD tissue by using a FACSAria flow cytometer. After cell sorting, labeling with GDF5 antibody, the cells were thereafter labeled with two different cell tracer dyes before application onto the IVD explants.

##### 

**Explant assay, small versus large cells (series 1)** Five rabbit IVDs collected from four animals (13 months of age) were minced, pooled, and placed in collagenase type II (Worthington Biochemicals, Lakewood, NJ, USA) for 24 hours in an incubator (37°C, 7% CO_2_, 93% air). The obtained single cells were pooled and washed with phosphate-buffered saline (PBS) before flow-cytometry analysis. The small (<10 μm cell diameter) and large (>10 μm cell diameter) cell populations were sorted from the total cell population with flow cytometry (see earlier). The small cells were thereafter labeled with carboxyfluorescein-diacetate-succinimidyl ester (CFDA-SE) cell-tracer kit (Invitrogen, Carlsbad, CA, USA), and the large cells were labeled with Cell tracker Orange CMRA (Invitrogen), according to the manufacturer’s instructions.

Eight IVDs were placed in cell-culture inserts in a six-well plate and cultivated in DMEM/F12 (Gibco, Grand Island, NY, USA) supplemented with 10% rabbit serum (collected and pooled from the same animals). A mix of fluorescence-labeled small (<10-μm cell diameter) and large cells (>10-μm cell diameter) was carefully pipetted on top of eight disc explants and allowed to adhere for 2 hours before the rest of the media was added. In addition, four IVD explants with large cells only and three IVD explants with small cells only were cultivated simultaneously.

Ten thousand labeled cells were pipetted onto the surface of each IVD explant. The cultured explants were harvested at days 2, 7, 12, and 16, and placed in 4% formaldehyde (Histolab, Sweden), and paraffin sections were prepared. The cultured explants were cut in a sagittal direction, and the depth of the deepest migrating cells was measured from the exposed IVD surface. A naive IVD served as negative control.

##### 

**Explant assay- GDF5^+^ cells (series 2)** Five IVDs from four animals (11 months of age) were minced, pooled, and placed in collagenase type II (Worthington Biochemicals) for 24 hours in an incubator (37°C, 7% CO_2_, 93% air). The obtained single cells were pooled and washed with PBS before flow-cytometry analysis. The GDF5-positive cells were sorted out from the total cell population by using a goat anti-GDF5 antibody (Santa Cruz Biotechnology Inc., Santa Cruz, CA, USA). The GDF5^+^ cells were thereafter labeled with CFDA-SE Vybrandt cell tracer kit (Invitrogen) according to the manufacturer’s instructions.

Seven intact IVDs were placed in cell-culture inserts in a six-well plate and cultivated in DMEM/F12 (Gibco) supplemented with 10% rabbit serum. The GDF5^+^ cells were carefully pipetted on top of the IVD explants and allowed to adhere for 2 hours before the rest of the media was added. The 10,000 labeled cells were pipetted onto the surface of each IVD explant.

##### 

**Detection of fluorochrome-labeled cells in series 1 and 2** In series 1, results were visualized by using a fluorescence microscope Nikon Equilopse80i (Nikon, Tokyo, Japan), and in series 2, confocal photos with Zeiss LSM 510 confocal microscope were collected of IVDs at 2, 5, 7, 12, and 16 days after initiation of cultures. The cultured explants were harvested, sectioned sagitally, cell nuclei stained with DAPI, and thereafter, and sections were covered with Prolong gold mounting media (Invitrogen).

#### ***Experiment B***

##### 

**Cellular migration studies, in vivo labeling with BrdU, tibia** Eleven rabbits, 3 months of age, were used in the experiments. Nine rabbits were exposed to BrdU (20 mg/kg body weight) diluted in the drinking water for 12 days. To monitor drinking rate per day, an exact dose of water was given to the animals every day. The rabbits were kept in separate cages during the BrdU administration and killed at 14, 28, and 56 days (counted from the first day of BrdU administration) with an overdose of methylphenobarbital (Apoteket AB, Sweden). Tibias were collected from each animal. Three control animals not exposed to BrdU served as negative controls. Control tissues from small intestines were used as positive controls for BrdU staining and for comparison with known stem cell niches for BrdU expression.

##### Immunohistochemistry detection of BrdU^+^ cells

Detection of BrdU-positive cells was performed by using an antigen-retrieval step with 10 m*M* citrate buffer, pH 6.0. Sections were incubated in 2 *M* HCl (Merck, Darmstadt, Germany) for 1 hour at 37°C, blocked with 2% bovine serum albumin (BSA; Sigma, St. Louis, MO, USA) and 0.3% Triton–X100 (Sigma Aldrich) diluted in phosphate–buffered saline (PBS), for 30 minutes at room temperature (RT). Mouse anti-BrdU antibody (DAKO, Glostrup, Denmark), dilution 1:250, was added, and sections were incubated for 12 hours at 4°C. Development was performed with secondary antibody goat anti-mouse, Alexa Fluor 546 (Invitrogen). 3 hours at RT. Nuclear staining was performed by using DAPI 0.05 μg/ml solution (4.6 diamino-2-phenylindole; Sigma) on all sections for 3 minutes, and samples were covered by using Prolong Gold antifade mounting media (Invitrogen). Results were visualized with a Nikon fluorescence microscope Eclipse 600 by using NIS-element software. Sections from tibia collected from non-BrdU-exposed animals served as negative controls and were analyzed by using the same protocol as described earlier.

Positive controls were sections from rabbit intestine collected from the BrdU-exposed rabbits. BrdU cells were counted in defined regions in KJs; articular cartilage, potential migration route (PMR), and potential niche region. The regions were chosen based on previous studies concerning the presence of stem cell niches (SNs) located in the zone of Ranvier groove in the KJ region adjacent to the epiphyseal plate [14,55] and previously described niche regions in the IVD area [29,42].

##### Immunohistochemistry: detection of SNAI1^+^ cells

Sections (KJs) from five animals were prepared for IHC, as described earlier, and investigated for SNAI1^+^ (cellular migration marker) cells. An antigen-retrieval step with 0.1 m*M* citrate buffer, pH 6.0, was performed, followed by blocking with 2% BSA. Goat anti-SNAI1 (Santa Cruz Biotechnology, Santa Cruz, CA, USA), dilution 1:100, was added to the sections, and the sections were incubated for 12 hours at 4°C. Secondary antibody; donkey anti-goat, dilution 1:250 Alexa Fluor 546 (Invitrogen) was added, and sections were incubated for 3 hours at RT in nuclei staining and mounting media, as described earlier. Isotype controls and/or sections with primary antibody omitted were used as negative controls.

#### ***Experiment C***

##### 

**In situ labeling with Fe nanoparticle cell tracer** The animals were anesthetized before surgery with intramuscular injections of fentanyl/fluanisone (HypnormVR; Janssen, Buckinghamshire, United Kingdom); 0.7 mg/kg body weight, and an intraperitoneal injection of diazepam (StesolidVR; Actavis AB, Sweden), 1.5 mg/kg body weight).

##### 

**Figure 3:**
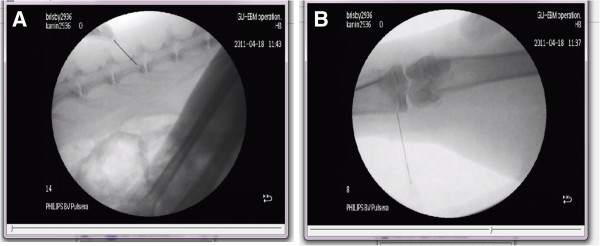
**Injections of cell tracer in IVDs and KJs into niche regions.** X-ray images showing injections of cell tracers (iron nanoparticles or CDFA-SE) in **(A)** the IVD niche region and **(B)** the niche region of KJs located adjacent to the epiphyseal plates (EPs). A 20-gauge needle was used for the injections of cell tracers.

**Tibias** Injections of 10 μl of 1 mg/ml cell tracer (Endorem; Braun Medical, Melsungen, Germany) (Fe nanoparticles) were given into the lateral region of the tibias adjacent to the epiphyseal plate (niche region; SN; right leg) under full visual control by using a 20-gauge needle (Figure 3). The right tibia was thereafter imbedded in paraffin, and sections (5 to 7 μm) were prepared. In each animal, the naïve tibia from the left leg served as control tissue.

##### 

**IVDs** The animals were anesthetized before surgery, as described. The cell-tracer compound (Fe nanoparticles) was injected into niche regions (SNs) of IVDs with a 20-gauge needle under full visual control. Injections of 10 μl 1 mg/ml Fe nanoparticles into the L2 to L3 level was performed under full visual control. Naive undisturbed IVDs served as negative controls (Figure 3).

The animals were sacrificed at 2 to 3 weeks (group 1) and after 5 to 6 weeks (group 2) with an overdose of methylphenobarbital (APL), and the lumbar IVDs (LI to LV) and KJs were collected.

##### 

**Histology of explants** The tibia and IVDs were consecutively sectioned (5- to 7-μm sections), serially numbered, and every tenth to eleventh section was stained for orientation with hematoxylin and for Fe-labeled cells by using standard protocols [56,57].

##### 

**Tracing of migrating Fe-labeled cells** Detection of Fe-labeled cells was performed in the sections by using standard Fe-staining protocols with the Prussian blue reaction (Mallory method) [57]. More than two observations of Fe-labeled cells per organ was considered a positive result for each animal.

## Results

Three main experiments were performed to investigate cellular migration in cartilaginous tissues of the IVDs and KJs: (a) an *in vitro* explants study (IVDs), (b *in vivo* labeling with BrdU (KJs), and *in situ* labeling with Fe particles in both IVDs and KJs (Figures 1, 2, and 3).

The outcome of the investigation of cellular migration in experiments A through C was as follows.

### Experiment A: *Ex vivo* study of migration in IVD explants

Flow-cytometry results of lapine IVDs were 64.4% small cells (<10-μm cell diameter), 23.7% large cells (>10-μm cell diameter), and 3.8% GDF5^+^ cells. Viability staining showed 96.5% viable and 3.5% nonviable cells.

**Figure 4:**
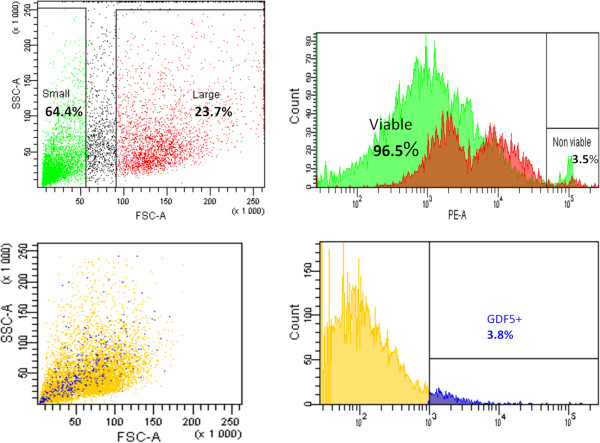
**Viability tests.** Upper row: 64.4% small cells (cell diameter <10 μm) and 23.7% large cells (cell diameter >10 μm) of the total IVD (AF and NP) cell population were sorted by size by using the forward-scatter parameter, and gating parameters are indicated by squares. Outcome of the test of viability was 96.5% viable and 3.5% nonviable cells. Lower row: 3. 8% GDF5^+^ cells were sorted from the total cell population by using an antibody directed toward GDF5.

**Figure 5:**
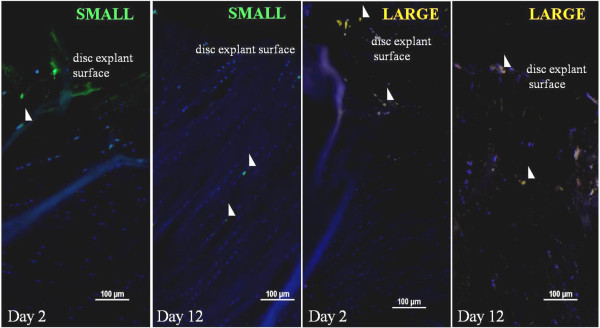
**Selected images of migrating fluorescence-labeled small (diameter <10 μm) and large (diameter >10 μm) rabbit IVD cells isolated with flow cytometry (AF and NP) placed on top of lapine IVD explants.** The two left images show migrating small cells (green) in explants at days 2 and 12, and the two right images show migrating large cells (orange) in explants at days 2 and 12 (white arrows).

**Figure 6:**
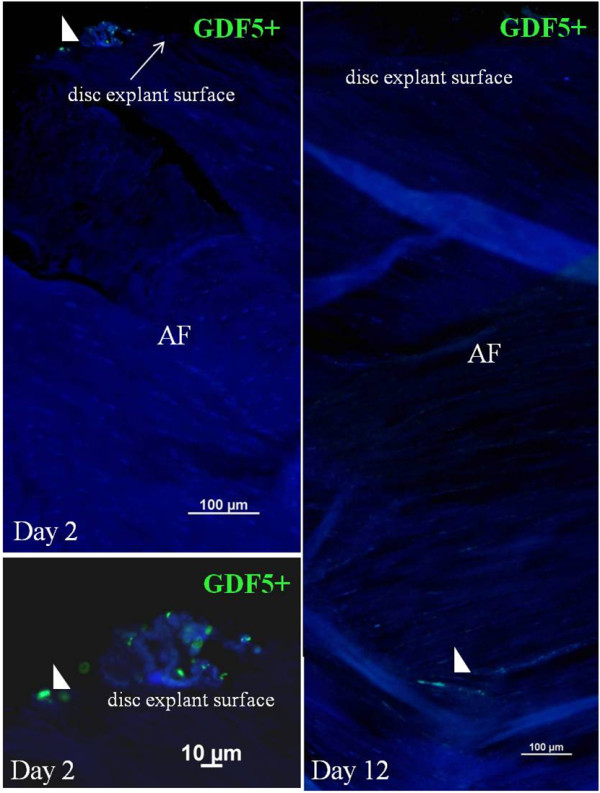
**Selected images of migrating GDF5**^**+ **^**cells (green) from lapine IVDs (sorted with flow cytometry) placed on top of lapine IVD explants and monitored after 2 and 12 days.** Nuclei stained with DAPI (blue).

**Figure 7:**
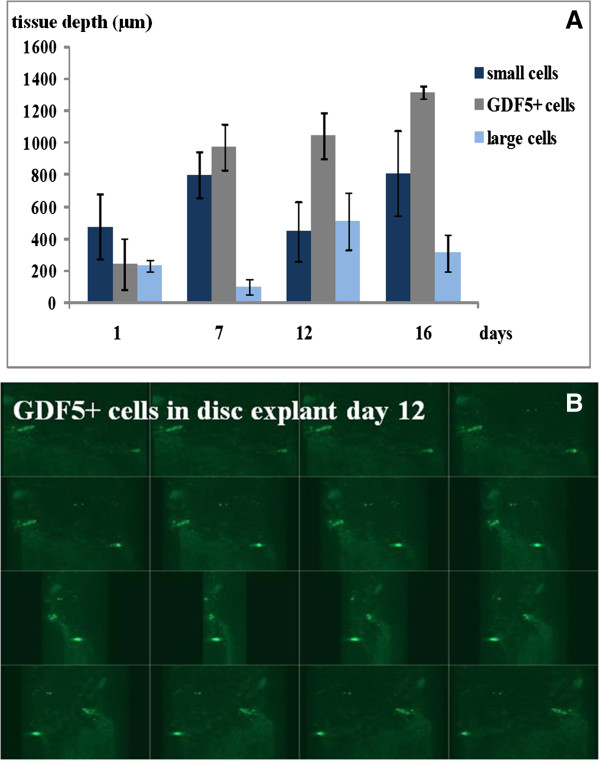
**Tissue-depth levels and distribution of migrating cells in rabbit IVD explants. (A)** Bar graphs displaying the deepest tissue level where different cell types were found after migration within lapine IVD explants. Mean values of four observations/IVDs seen at different parts of the IVD (AF). Small cells are displayed as dark-blue bars, GDF5^+^ cells as grey bars, and large cells as light-blue bars. **(B)** A 3D confocal image illustrating the GDF5cell distribution visible as light-green dots in a lapine IVD explant at day 12. Magnification: ×200.

In IVD explants in *in vitro* cultures, migrating small cells were labeled with CDFA and large cells (>10 μm), Orange CMRA labeled, were observed. Small cells showed a greater capability to migrate through the AF of the IVD explants compared with large cells. GDF5^+^ cells and ≤10-μm cells displayed better migration capability in IVD explants compared with large cells examined by migration depth. GDF5^+^ cells were detected as deep as 1,300 μm (measured from the surface of the explant) after 16 days (Figures 4, 5, 6, and 7).

### Experiment B: cellular-migration studies *(in vivo* labeling with BrdU) in KJ (tibia)

**Figure 8:**
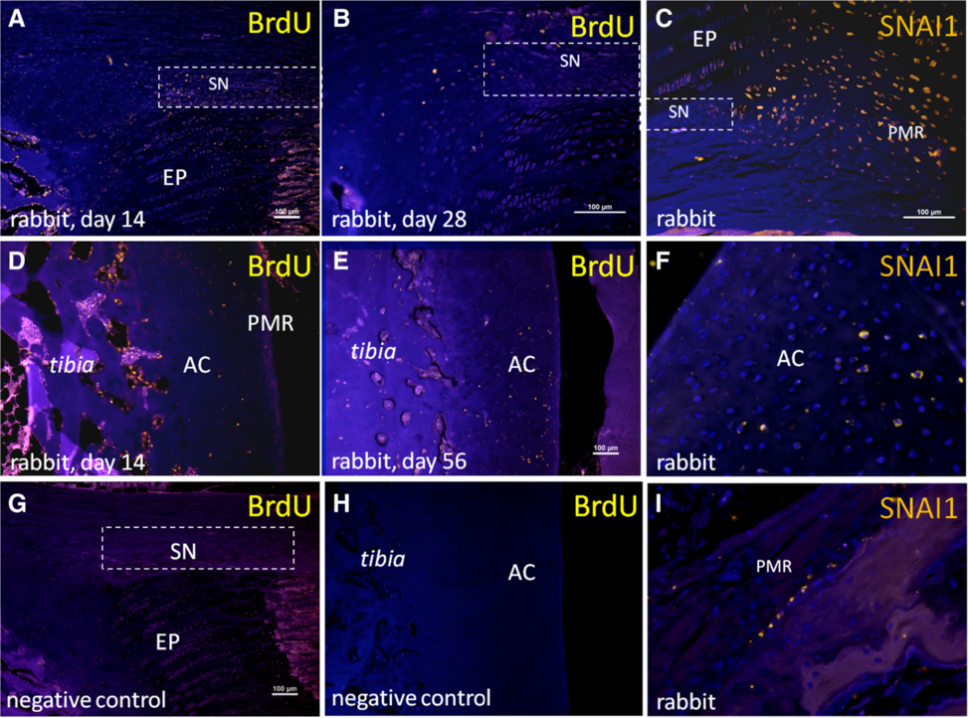
**Selected images of BrdU**^**+ **^**cells (yellow) in investigated KJ regions. (A)** BrdU^+^ cell in niche region (SN) at day 14. **(B)** BrdU^+^ cells predominantly just outside niche region (SN) at day 28. **(C)** SNAI1^+^ cells (yellow) just outside niche region and in potential migration route (PMR) (note the larger fraction of SNAI1^+^ cells compared with the BrdU-labeled fraction of cells in Figure 6B). **(D)** Articular cartilage (AC) at day 14. **(E)** Articular cartilage (AC) at day 56**. (F)** SNAI1^+^ cells in AC. **(G)** Negative control, BrdU; niche region (SN). **(H)** Negative control, BrdU. AC. **(I)** SNAI1^+^ cells (yellow) in PMR. **(J)** BrdU^+^ cells in niche (SN) and PMR and BrdU^+^ cells in PMR. (Images J and K are merged from three separate photos). Nuclei stained with DAPI (blue).

**Figure 9:**
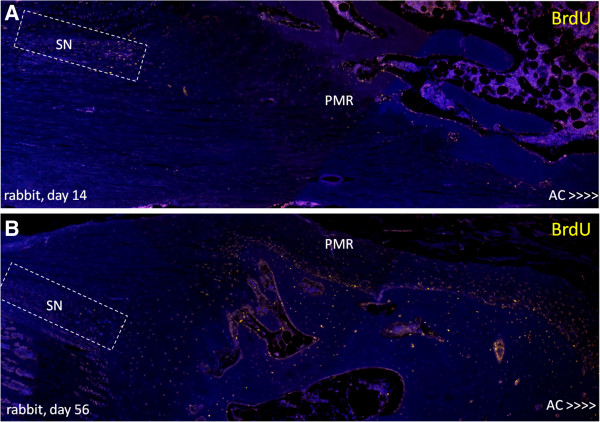
**Images (each image merged from three separate photos) of BrdU**^**+ **^**cells (yellow) in niche region (SN; square) and potential migration route (PMR) at the time points. (A)** 14 days and **(B)** 56 days in lapine tibia. Nuclei stained with DAPI (blue). Note the decrease in number of BrdU^+^ cells in SN and the more abundant BrdU^+^ cells in PMR at day 56 compared with day 14.

**Figure 10:**
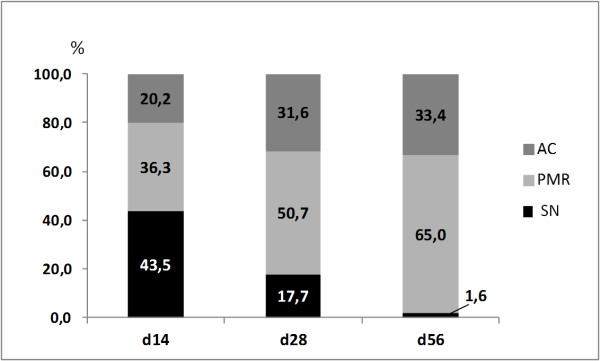
**Bars in figures represent the percentage of BrdU**^**+ **^**cells in AC (grey), the PMR regions (light grey) that originate from the niche (SN) (black).** The mean number of cells for each localization and time point was used in the calculations. Note the more-pronounced shift of BrdU^+^ cells detected in the PMR (potential migration route) over time, which indicate migration of BrdU^+^ cells originating from the niche (SN) into this area.

BrdU^**+**^ cells were observed at early time points (14 days) in niche regions of KJs, at later time points (28 and 56 days) in a potential migration area (PMR), and in articular cartilage (AC), indicating a gradual migration over time of cells from niches toward the AC. Negative controls showed no staining (Figures 8, 9, and 10).

#### ***Detection of SNAI1***^+^ ***cells in KJ***

SNAI1^+^ cells were detected both along the PMR, just adjacent to the niche border zone, and in the superficial zone of AC (four of five animals), a few cell layers from the AC surface. In AC, only a few solitary SNAI1^+^ cells were detected. No SNAI1^+^ cells were detected within the niche (Figure 8).

### Experiment C: *In situ* Fe- labeling of cells in niches

#### ***In vivo, in situ labeling with cell tracer in IVD and KJ***

**Figure 11:**
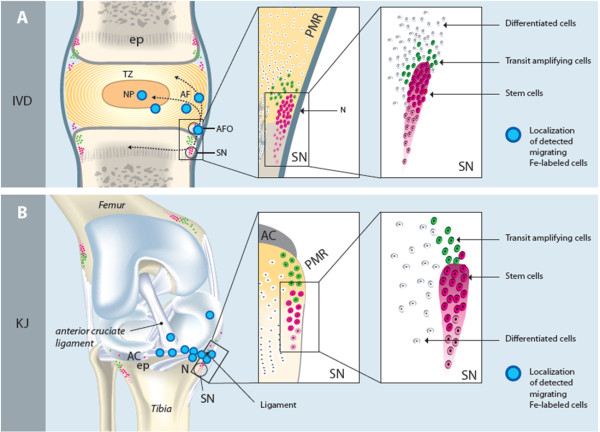
**Hypothetical schematic image that displays the investigated areas. (A)** IVD; the niche regions (SNs; just lateral to the epiphyseal plate), and the annulus fibrosus (AF), AF outer region (AFo), and the potential migration route (PMR, area between niches (SN) and the IVD). In **(B)** KJ; the niche regions (SN) of (just lateral to the epiphyseal plate) and the articular cartilage (AC) and the potential migration route (PMR, area between niches (SN) and the AC). Detection of Fe-labeled cells in different areas is marked with blue dots.

Cells *in vivo* labeled with the cell tracer (iron nanoparticles, IVD and KJ) were found in comparable regions and levels in the animals; located between niches (SN; lateral to the epiphyseal plate), and the annulus fibrosus outer region (AFo) and in articular cartilage (AC) of the KJ, the potential IVD migration-route region (PMR). Hypothetical potential cellular migration route (PMR) in IVD and KJ regions and localizations of detected Fe-labeled + cells are illustrated in Figure 11.

##### 

**Detection of Fe^+^ cells in IVDs** In the IVD region, Fe^+^ cells were detected in all animals. In AF, Fe^+^ cells were detected 11 of 12 animals; at 2 to 3 weeks (group 1) in three of three animals, and at 5 to 6 weeks (group 2) in eight of nine animals as solitary cells. In NP, Fe^+^ cells were found in only two of 12 animals (at 2 to 3 weeks (group 1) and 5 to 6 weeks (group 2). In the PMR region of the IVD, Fe^+^ cells were detected in three of 12 animals; at 2 weeks (group 1), in one of three animals, and at 5 to 6 weeks (group 2) in two of nine animals.

**Figure 12:**
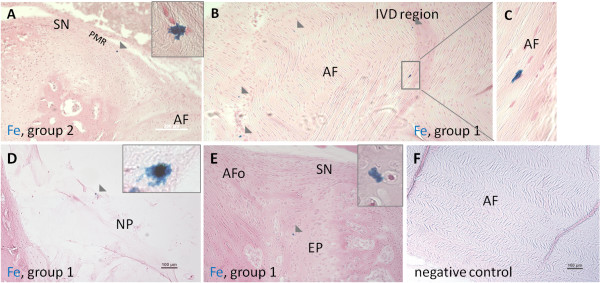
**Images of Fe-labeled cells (blue) in investigated intervertebral disc (IVD) regions.** Images from group 1 animals (2 to 3 weeks) and group 2 animals (5 to 6 weeks) after *in situ* Fe-labeling of cells in the niche region (SN). **(A)** Potential migration route (PMR) region. **(B)** The annulus fibrosus (AF). **(C)** Enlarged image of iron-labeled cell in AF indicated by square. **(D)** The nucleus pulposus (NP). **(E)** The germinal zone of the epiphyseal plate (EP). **(F)** Negative control; AF. Note the elongated migratory phenotype of cells in B and C. Enlarged images of Fe-labeled cells are enclosed in corners of images. Fe-labeled cells (blue) are indicated by grey arrows. Staining: russian blue reaction (Mallory method).

The Fe^+^ cells were detected mainly in outer AF lamellae. In AF, some of the detected cells were located in interspaces between the AF lamellae and aligned in the same angle as AF in the lamellae; Figures 11 and 12.

##### 

**Detection of Fe^+^ cells in KJs** In KJ (tibias), Fe^+^ cells were detected in 11 of 12 animals in the different investigated regions: AC, PMR, and niche regions (SNs).

In AC, Fe^+^ cells were detected six of nine animals; at 2 to 3 weeks (group 1) in two of three animals, and at 5 to 6 weeks (group 2) in four of nine animals as solitary cells, found at different tissue depths, in different cell-layer zones; in the superficial and middle zones of AC. Solitary, Fe^+^ cells also were found in six of 12 of the animals distributed in different locations in the PMR region; at 2 weeks (group 1) in one of three animals, and at 5 to 6 weeks (group 2) in five of nine animals (Figures 11 through 13.

**Figure 13:**
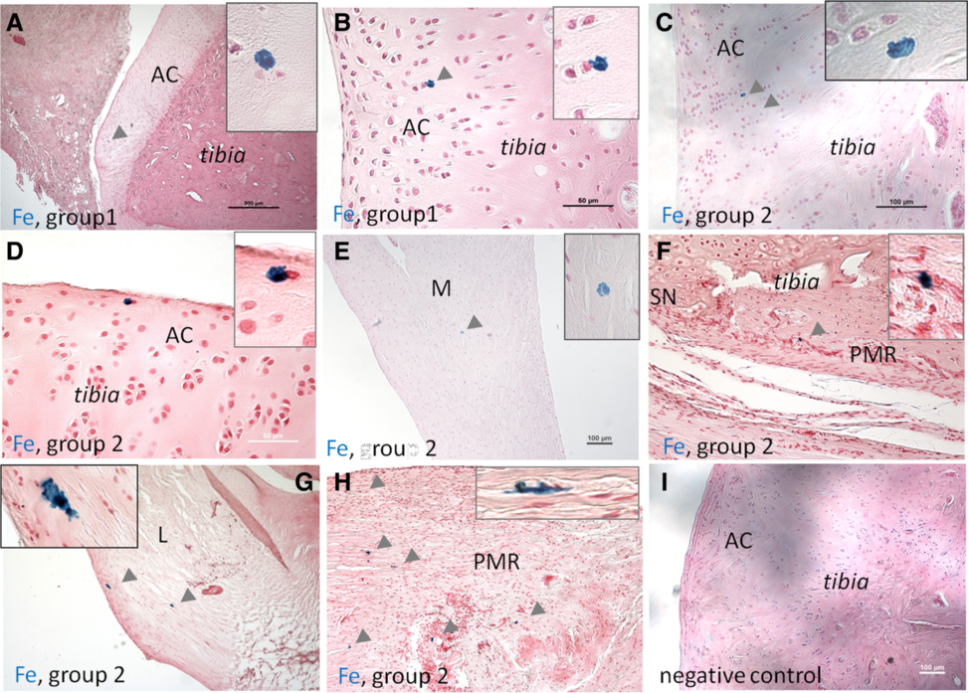
**Images of Fe-labeled cells (blue) in investigated knee-joint (KJ) regions.** Images of Fe-labeled cells (blue) from group 1 animals (2 to 3 weeks) and group 2 animals (5 to 6 weeks) after *in situ* Fe labeling of cells in the niche region (SN) in investigated knee joint (KJ) regions **(A through D)** in articular cartilage (AC). **(E)** in *meniscus* (M), **(F)** in potential migration route (PMR), **(G)** in cruciate ligament (L), **(H)** in the PMR region, and in **(I)** negative control; AC. Note the elongated migratory phenotype of cells in (G) and (H). Enlarged images of Fe-labeled cells are enclosed in corners of images. Fe-labeled cells (blue) are indicated by grey arrows. Staining: russian blue reaction (Mallory method).

No Fe^+^ cells were detected within the niche regions (SNs) of IVDs or KJs at any time.

Furthermore, a few observations of solitary Fe^+^ cells were also made in adipose, the germinal zone of epiphyseal plate, cruciate ligaments, and in meniscus tissues in some of the animals. Negative controls showed no staining (Figures 11, 12, and 13).

## Discussion

The results obtained in this study demonstrate the capability of cells to migrate through ligaments and fibrous tissue from niches toward and into cartilaginous tissues. In this study, we observed cell migration in two different lapine cartilaginous tissue types: in the intervertebral disc region (fibrocartilage) and in the knee joint (articular cartilage/hyaline cartilage). In the IVD region, during embryogenesis, the notochord is gradually surrounded and replaced by immigrating mesenchymal cells, which synthesize the fibrocartilaginous AF and the vertebral bodies [58,59].

The IVD explant experiments (experiment A) show that cells are capable of migration within the IVD tissue and seem to follow the intercellular space direction of the lamellae (collagen I-rich fibers) of the AF (Figure 5). The sorted cell population of the small cells (≤10 μm diameter) displayed a better migration capability compared with larger cells (>10 μm diameter) and most likely represent a prechondrocytic cell population that may participate in normal growth and regeneration of the knee joint and the IVD. Presumably, small cells are capable of migrating more efficiently through tissues compared with larger cells because of the narrow intercellular spaces. Typical stem cell features are small cellular size and good migration capability. This is supported by migration to a similar tissue depth by the GDF5^+^ cells (which are small). Other bone morphogenic proteins, such as -2, -4, and -7, have been observed to stimulate chemotactic migration of human mesenchymal stem cells *in vitro* (Boyden chamber model) [60,61]. Stem cell niches have been proposed both in the intervertebral disc region adjacent to the epiphyseal plate (EP) and in the outer border of AF [26] and in the knee joint in the zone of Ranvier groove [14,55].

Cells of different morphology have been observed within the zone of Ranvier groove, and in the center of the groove, the cells are more densely packed. This area of densely packed cells has been demonstrated to harbor stem cells/progenitor cells [14,29,30,42]. The ring of LaCroix, a fibrous tissue layer surrounding this groove, has also been suggested to contain precartilaginous cells; a niche and serve as a suppliant reservoir of cells for the germinal zone of the EP by Fenichel et al. [28,33]. The EP and an intact perichondrial zone are crucial for normal growth of the long bones [31]. Injuries in childhood, in the zone of Ranvier groove and in the EP (for example, Salter-Harris type IV fractures) can result in severe skeletal growth anomalies [32,33].

In experiment B, a shift in number of BrdU^+^ cells in different locations with time (in relation to the incorporation period) indicate migration of slow-cycling cells (label-retaining cells). The direction of migrating BrdU^+^ cells was from the niche toward the articular cartilage of the KJ (present study) and the IVD [29].

Some of the observed cells in experiment C clearly demonstrated a typical phenotype associated with migration: elongated cellular shape and cellular protrusions [48,62]. This was observed both in the IVD region and in the knee joint (Figures 12 and 13). The expression of SNAI1, which is involved in the rearrangement of cytoskeletal elements during migration, further supports cellular migration in the investigated areas. Furthermore, the alignment of collagen fibrils has been shown to be important for cellular migration (for example, in human breast carcinoma [63,64]).

The findings in this study are in alignment with a previous study in osteoarthritic cartilage in the KJ, where chondroprogenitor cell migrating capacity was demonstrated in a murine *in vitro* explant model [65]. Minor injuries in the articular cartilage of the KJ in young (skeletally immature) animals have been demonstrated to heal, whereas larger injuries exhibit a poor or no self-regeneration capacity [66,67]. Articular cartilage in the KJ contains populations of progenitor cells [12,15,62]. Further, human articular chondrocytes cultured *in vitro* were demonstrated to display phenotypic plasticity with chondrogenic, adipogenic, and osteogenic potential [14,61].

Similar to the KJ, in an *in vitro* organ-culture study (murine epiphyseal fracture model), a cellular migration pattern was described from the epiphyseal plate into the IVD, and the migrating cells were classified as chondrocytes [68]. However, the phenotype of these cells was not completely described. Additionally, a few observations have been made of minor regeneration processes within the IVD, especially in the outer region of the AF [69-72]. Additionally, the presence of progenitor cells has been described in the human IVD [73,74], and the possibility of influencing the proliferation and differentiation of human degenerated IVD cells *in vitro* was recently reported [16].

Previously, increased cell proliferation and cell migration in the IVD region [75], in the KJ [76], and in tendon [77], as an effect of physical exercise (treadmill), was observed in animal models (murine models), which indicates that cell proliferation and migration can be stimulated.

## Conclusions

The results from this study suggest that the niche regions in KJs and IVD and their cellular activity (for example, cellular migration) are important for growth mechanisms of cartilage, tissue maintenance, and possibly the regeneration of cartilaginous tissues in adult mammals. In the IVD and KJ regions, increased knowledge about local immature cells and cellular interactions with different biomaterials can be of interest for tissue engineering and/or potential stimulation of local progenitor cells *in situ* when developing biologic treatment strategies for disc-degeneration disease and osteoarthritis.

## Abbreviations

AC: Articular cartilage; AF: Annulus fibrosus; AFo: annulus fibrosus outer region; BrdU: 5-bromo-2-deoxyuridine; IVD: Intervertebral disc; KJ: Knee joint; NP: Nucleus pulposus; PMR: Potential migration route; SN: Stem cell niche.

## Competing interests

The authors declare that they have no competing interests.

## Authors’ contributions

HBH, HB, and AL conceived of and designed the research experiments. HBH performed the cell cultures and animal experiments, immunohistochemistry/microscopy analyses, interpretation of results, and drafted the manuscript, AL provided financial support, interpretation of results, and manuscript writing. ES advised on interpretation of results, and participated in *in vitro* experiments with explant cultures and in manuscript writing. KJ carried out the flow-cytometry analyses and advised on interpretation of data. CT provided technical assistance and expertise in the fluorescence confocal microscopy analyses and interpretation of data. JM provided technical assistance in the fluorescence confocal microscopy analyses and expertise regarding cellular migration. HB provided financial support, interpretation of results, manuscript writing, and animal experiments. All authors read and approved the final manuscript.
